# Ca^2+^-imaging and photo-manipulation of the simple gut of zebrafish larvae in vivo

**DOI:** 10.1038/s41598-022-05895-4

**Published:** 2022-02-07

**Authors:** Shin-ichi Okamoto, Kohei Hatta

**Affiliations:** Graduate School of Science, University of Hyogo, 3-2-1 Kouto, Kamigori, Ako-gun, Hyogo, 678-1297 Japan

**Keywords:** Zebrafish, Feeding behaviour, Neurophysiology

## Abstract

Zebrafish larval gut could be considered as an excellent model to study functions of vertebrate digestive organs, by virtue of its simplicity and transparency as well as the availability of mutants. However, there has been scant investigation of the detailed behavior of muscular and enteric nervous systems to convey bolus, an aggregate of digested food. Here we visualized peristalsis using transgenic lines expressing a genetically encoded Ca^2+^ sensor in the circular smooth muscles. An intermittent Ca^2+^ signal cycle was observed at the oral side of the bolus, with Ca^2+^ waves descending and ascending from there. We also identified a regular cycle of weaker movement that occurs regardless of the presence or absence of bolus, corresponding likely to slow waves. Direct photo-stimulation of circular smooth muscles expressing ChR2 could cause local constriction of the gut, while the stimulation of a single or a few neurons could cause the local induction or arrest of gut movements. These results indicate that the larval gut of zebrafish has basic features found in adult mammals despite the small number of enteric neurons, providing a foundation for the study, at the single-cell level in vivo*,* in controlling the gut behaviors in vertebrates.

## Introduction

The gut is an important organ for digestion and the absorption of nutrients and water. The bolus, once swallowed, is conveyed through the gut to the anus. In vertebrates, this movement, peristalsis, is achieved by two sets of muscular systems. These systems are regulated by neuronal groups of the enteric nervous system (ENS), which is derived from the neural crest^1^. In mammals, the intestine is lined by distinct concentric layers: the mucosa (the epithelium that forms the innermost layer), subjacent connective tissue, and thin layer of smooth muscle (inner circular and outer longitudinal smooth muscles). A part of the ENS, the myenteric plexus exists between the longitudinal and circular muscles and provides motor innervations to both layers in the tunica muscularis, while the submucosal plexus innervates cells in the epithelial layer and the smooth muscle of the muscularis mucosa^2^. The human ENS contains 100 million neurons. The ENS contains its own sensory and motor system and can cause various movements, such as the peristaltic reflex, even if the gut is isolated from the body^2^. The ENS, known as the “second brain”, is the only nervous system that can act independently from the central nervous system (CNS). In mammalian ENS, serotonin (5-HT) and acetylcholine are major excitatory transmitters, and nitric oxide (NO) generally has an inhibitory effect on smooth muscle cells^3–6^. Pacemaker cells (ICCs, interstitial cells of Cajal) have been reported to produce ‘slow waves’ propagating from the anterior to the posterior. ICCs are thought to be the main target of motoneurons, and they regulate smooth muscles via gap junctions^7,8^.

Although the zebrafish gut closely resembles the mammalian gut, the former is much simpler. Enteric neuronal cell bodies form only a single layer at a myenteric plexus without forming ganglia scattered on a single tubular plane^9^. Using Hu immunohistochemistry, about 380 enteric neuron cell bodies per wild-type intestine were identified at 5 days post-fertilization (dpf)^9^ and about 700 were identified at 8 dpf (our unpublished observation). Zebrafish gut has been reported to have many features in common with mammalian gut. The intrinsic enteric innervation of the zebrafish guts shows the expression of a number of neurochemicals, as in mammals^10,11^. Mutants that show a reduction of neurons in the ENS and abnormal motility have been isolated^12^. Despite these observations, the movement of the gut to convey the bolus has not been investigated in details. We thus studied gut movement by Ca^2+^ imaging^13^ and optogenetics^14,15^.

## Results and discussions

**Figure 1:**
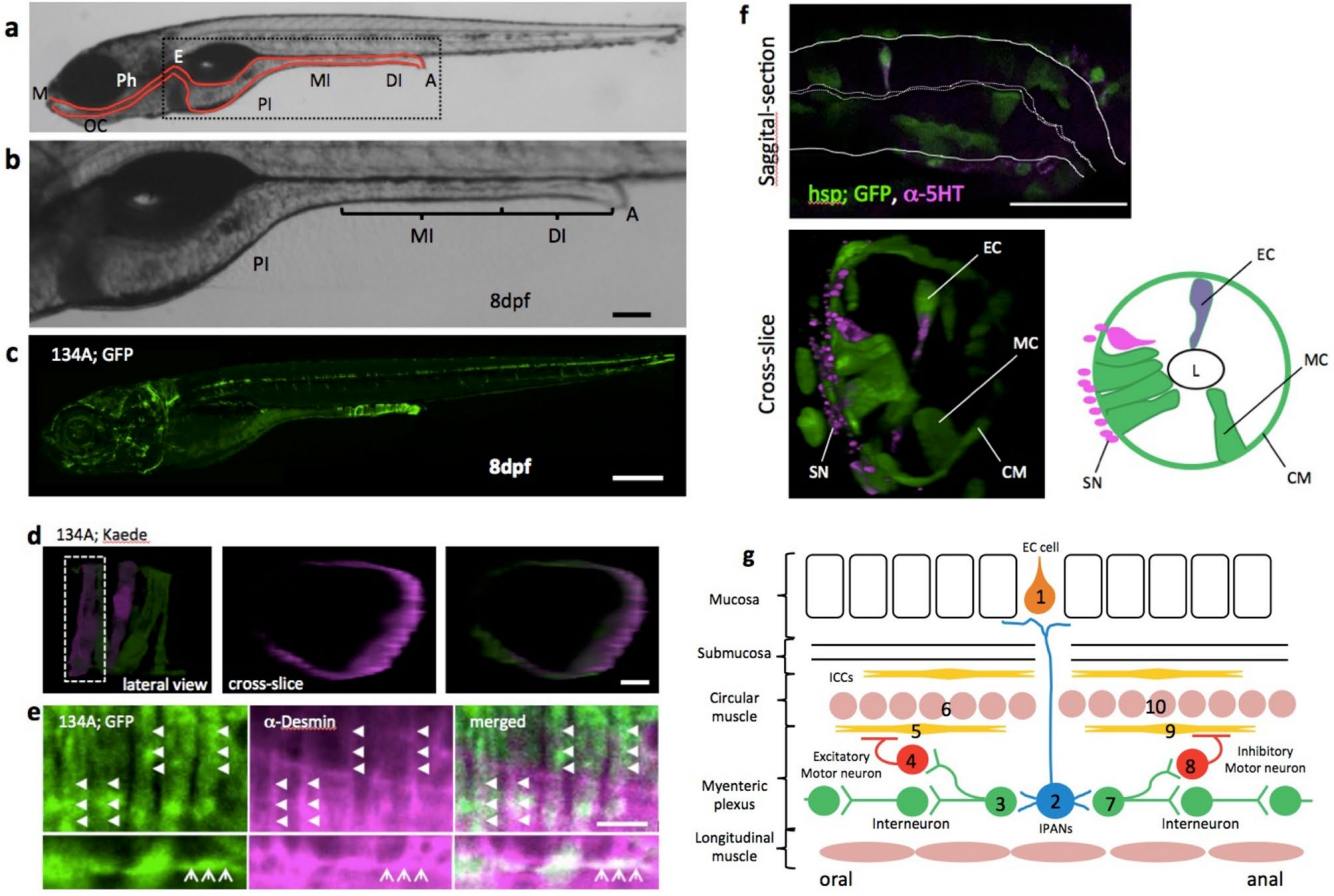
Simple structure of the zebrafish gut. (**a**) Lateral view of zebrafish larva at 8 dpf, indicating the principal digestive tract. The intestine is divided into three parts: proximal intestine (PI), middle intestine (MI), and the distal part of the intestine (DI)^10^. The PI is also referred to as the intestinal bulb and is thought to have similar functions as the stomach, which zebrafish do not have. The MI is thought to absorb nutrients and plays a role in mucosal immunity. The DI is analogous to the colon. *M* mouth, *OC* oral cavity, *Ph* pharynx, *E* esophagus, *PI* proximal intestine, *MI* middle intestine, *DI* distal intestine, *A* anus. (**b**) A higher-magnification image of the area indicated by the square in (**a**). Scale bar, 50 μm. (**c**) Lateral view of SAGFF(LF)134A; Tg(UAS: GFP) at 8 dpf. Scale bar, 200 μm. (**d**) Live confocal image of individual circular smooth muscles visualized by green to red photoconversion of Kaede fluorescence in SAGFF(LF)134A; Tg(UAS: Kaede) at 5 dpf. Lateral projection view shows two muscles photoconverted. Left side view. Cross-slice view of the dotted area shows a single red muscle. Scale bar, 10 μm. (**e**) Saggital sections of a confocal image of SAGFF(LF)134A; Tg(UAS: GFP) at 8 dpf stained with anti-Desmin antibody. Upper panels demonstrate Desmin filaments (in magenta, shown by triangles) located inside of each circular smooth muscles expressing GFP. Lower panels show an example of occasional appearance of longitudinal muscle cell expressing GFP, containing Desmin filaments (arrows). Scale bar, 10 μm. (**f**) Saggital section and cross-slice of the gut, indicating a variety of cell types visualized at 5 dpf in Tg(hsp70: Gal4); Tg(UAS: GFP) by heat shock at 3 dpf. Double immunostaining for anti-GFP (green) and anti-5-HT (magenta). *EC* serotonergic endochromaffin cells, *MC* mucosal cells, *L* lumen, *CM* circular muscle, *SN* neurites of serotonergic neurons. Scale bar, 50 μm. (**g**) Schematic diagram showing the hypothetical circuitry for the peristaltic reflex in the zebrafish gut. 1, endochromaffin cell; 2, intrinsic primary afferent cell; 3, ascending interneuron; 4, excitatory motoneuron; 5, 9, interstitial cells of Cajal; 6, 10, circular smooth muscles; 7, descending interneuron; 8, inhibitory motoneuron.

In double transgenic fish, SAGFF(LF)134A; Tg(UAS: GFP), GFP was expressed in the gut at the distal intestine and the posterior end of the middle intestine after 2 dpf^16^ (Fig. 1a–c). We found that GFP-positive cells were immunolabeled with anti-Desmin antibody, a marker for smooth muscles^9^ (Fig. 1e). We discerned a single cell by using a photoconversion technique^17^ with SAGFF(LF)134A; Tg(UAS: Kaede) in 5 dpf larvae. When we irradiated a single cell with a 405 nm laser, it was successfully labeled with red fluorescence in the local area (Fig. 1d). The photoconverted cell was ribbon-shaped, i. e., elongated and flat; it wrapped around the gut in perpendicular to the axis of the gut and had tapered ends (Fig. 1d). These data indicate that expression was mainly in the circular smooth muscles, while expression was also observed in some larvae in a small number of longitudinal muscles (Fig. 1e, lower panel).

**Figure 2:**
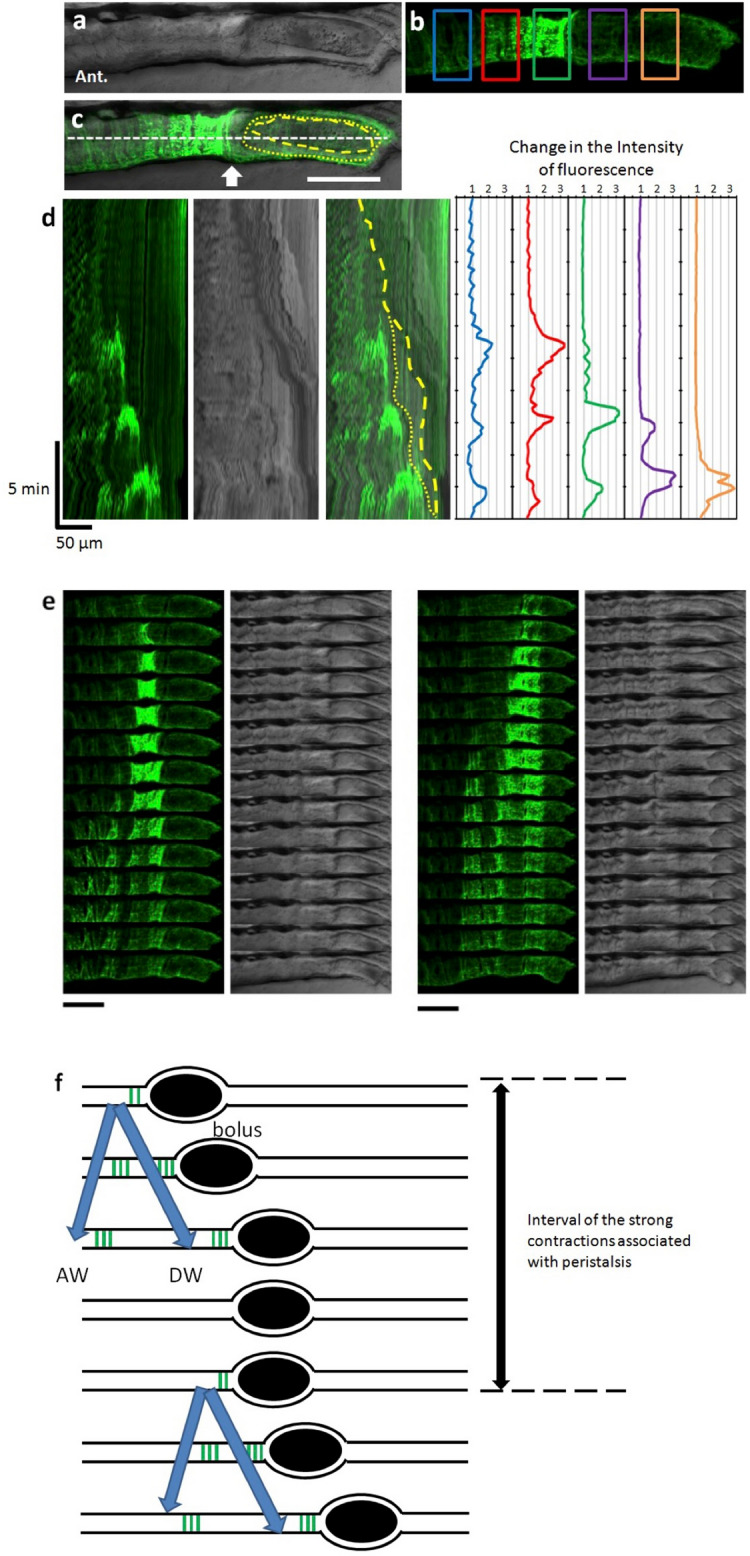
Peristaltic reflex visualized by Ca^2+^ imaging of circular smooth muscles. (**a–c**) Live confocal image of the lateral view of SAGFF(LF)134A; Tg(UAS: GCaMP3) at 8 dpf. Bright field image (**a**), GCaMP3 fluorescence (green) (**b**), and merged image (**c**). In (**b**), measurement locations are shown in rectangles. In (**c**), the yellow dashed line represents the outline of the bolus. The yellow dotted line represents the outline of the liquid surrounding the bolus. Scale bar, 50 μm. (**d**) Kymograph of GCaMP3 fluorescence, bright field image and merged image at the site noted by the white dashed line in (**c**), and time course of the fluorescence intensity at each site noted by the five squares in (**b**). (**e**) Time-lapse series of the second (left) and third (right) events in (**d**). Scale bars, 50 μm. Interval, 11 s. (**f**) Schematic diagrams showing Ca^2+^ events in the circular muscles at the peristaltic movement. Black ellipses denote bolus. Also see Supplementary video 1.

**Figure 3:**
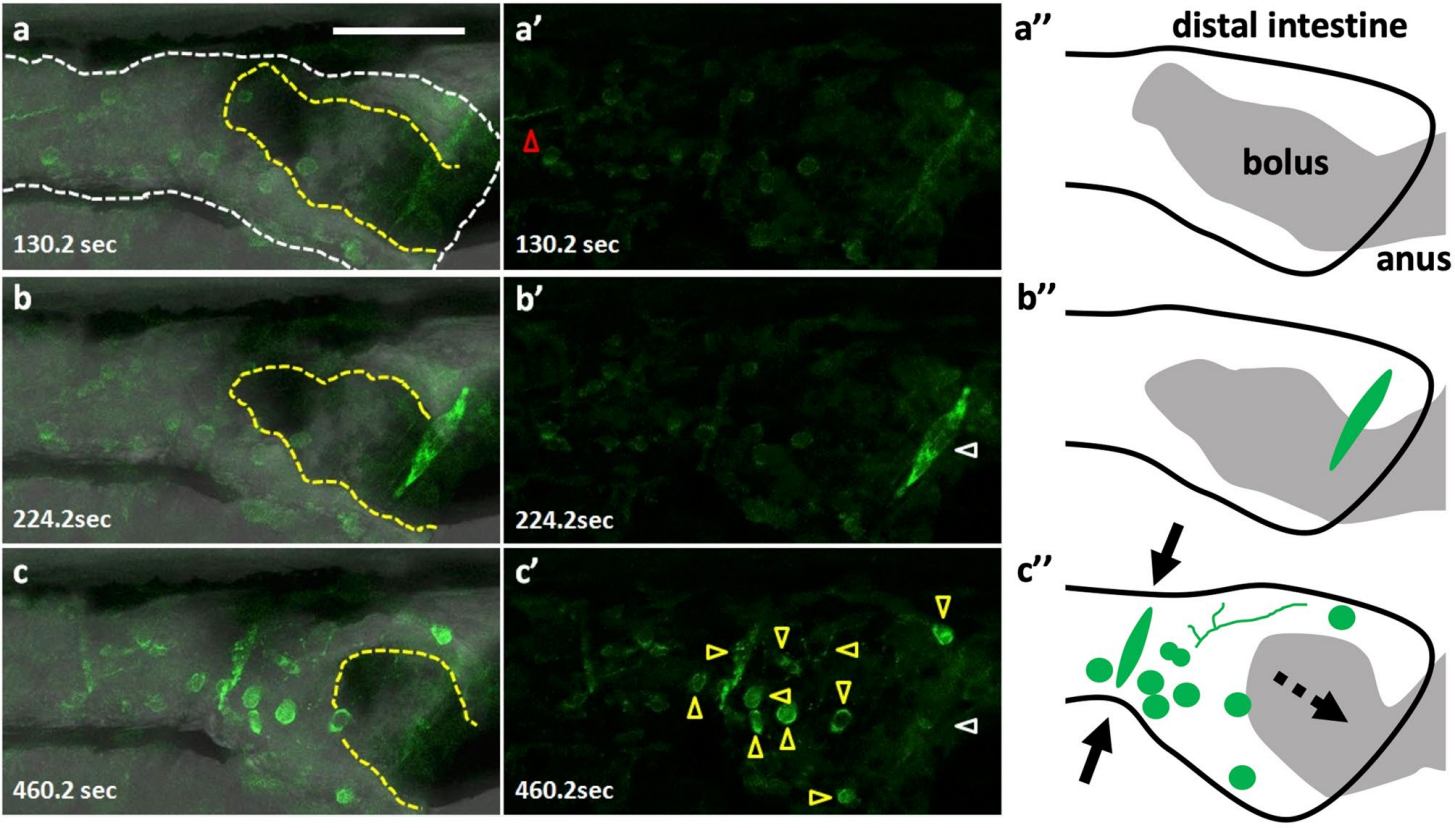
Peristaltic reflex visualized by Ca^2+^ imaging of a variety of cell types including putative enteric neurons and circular smooth muscles. Live images of putative enteric neurons and circular smooth muscles expressing GCaMP3 at 8 dpf in Tg(hsp70: Gal4); Tg(UAS: GCaMP3), heat-shocked at 6 dpf. The passage of the bolus was blocked at the anus by agarose gel, in an artificial condition of constipation. (**a–a’’**) the phase when most of the activity is arrested but an activated fiber with a distinct rhythm extends from the oral side (red triangle). (**b–b’’**) the phase when only the putative circular muscles at the anal side (white triangle) located on the bolus (yellow dashed lines) are activated. (**c–c’’**) the phase when the putative circular muscles located at the oral side of the bolus are activated together with most of the putative neurons and axons in the view (yellow triangles) (**a**–**c**), merged image; (**a’–c’**), fluorescence; (**a’’–c’’**), schematic drawings. Solid arrows indicate a contraction of the gut and a dashed arrow, the movement of the bolus. Lateral views. Anterior to the left. Scale bar, 50 μm. Also see Supplementary video 2.

We first visualized circular smooth muscle activity using the genetically encoded Ca^2+^ sensor GCaMP3^18,19^. To monitor changes in the intracellular Ca^2+^ concentration in circular smooth muscles of the gut during peristaltic movement, we fed larvae with paramecium from 5 dpf and performed Ca^2+^ imaging of the gut at 8 dpf of SAGFF(LF)134A; Tg(UAS: GCaMP3) from the lateral side. We found strong Ca^2+^ events in the circular smooth muscles during peristaltic movement in 7 out of 10 larvae. Figure 2a–c is a live confocal image of the lateral view. The fluorescence intensity of GCaMP3 increased in the contracted circular smooth muscles. Figure 2b shows representative examples of Ca^2+^ events (Supplementary video 1). Figure 2d shows kymographs of the GCaMP3 fluorescence and a bright field image, as well as the time course of intensity at each site, denoted by the five squares in Fig. 2b. The fluorescence intensity was stronger at the oral side of the bolus and weaker at the anal side. There was often a sharp boundary between the two sides (arrow in Fig. 2c). Strong Ca^2+^ events occurred repeatedly, typically starting at the oral side of the bolus. These Ca^2+^ events moved to the anal side (a movement tentatively called a descending wave: DW) and spread to the oral side (ascending wave: AW). In some cases AW was not obviously observed, especially when two or more boluses were in close proximity to each other. DW occurred along with propulsion and expulsion of the bolus. On the other hand, AW may reinforce the power that propels the bolus by adding more area of contraction on the oral side. Most samples showed DW (14/22 events, 7 larvae) and AW (14/22 events, 7 larvae) contractions. When the passage of the bolus was blocked at the anus by agarose gel, in an artificial condition of constipation, we found repeated strong Ca^2+^ events (Fig. 3, Supplementary video 2). We calculated the duration and interval of each Ca^2+^ event (Fig. 2e,f). The duration (from 50% rise to 50% decay) of the Ca^2+^ events ranged from 9.6 to 106.2 s with a mean ± standard deviation (s.d.) of 40 ± 22 s (n = 87, 7 larvae). The mean interval between Ca^2+^ events at the oral side of the bolus was 175 ± 105 s (n = 15, 4 larvae).

The activity of other cell types associated with gut movement was also investigated by using Tg(hsp70: Gal4)^20^; Tg(UAS: GCaMP3) (Fig. 3, Supplementary video 2). Heat shock at 6 dpf induced the mosaic expression of GCaMP3 at 8 dpf in a variety of cell types. The cells that have a round soma and long processes are putative enteric neurons, the cells elongated perpendicularly with gut axis are putative circular smooth muscles. Time-lapse observation was also performed on the larva whose bolus was blocked by agarose. We identified two phases of activity in which a population of neurons was synchronously activated or inactivated with muscles involved in peristaltic movement (Fig. 3). Further investigation is needed to identify the neuronal types or other cell types and their connections in the ENS.

**Figure 4:**
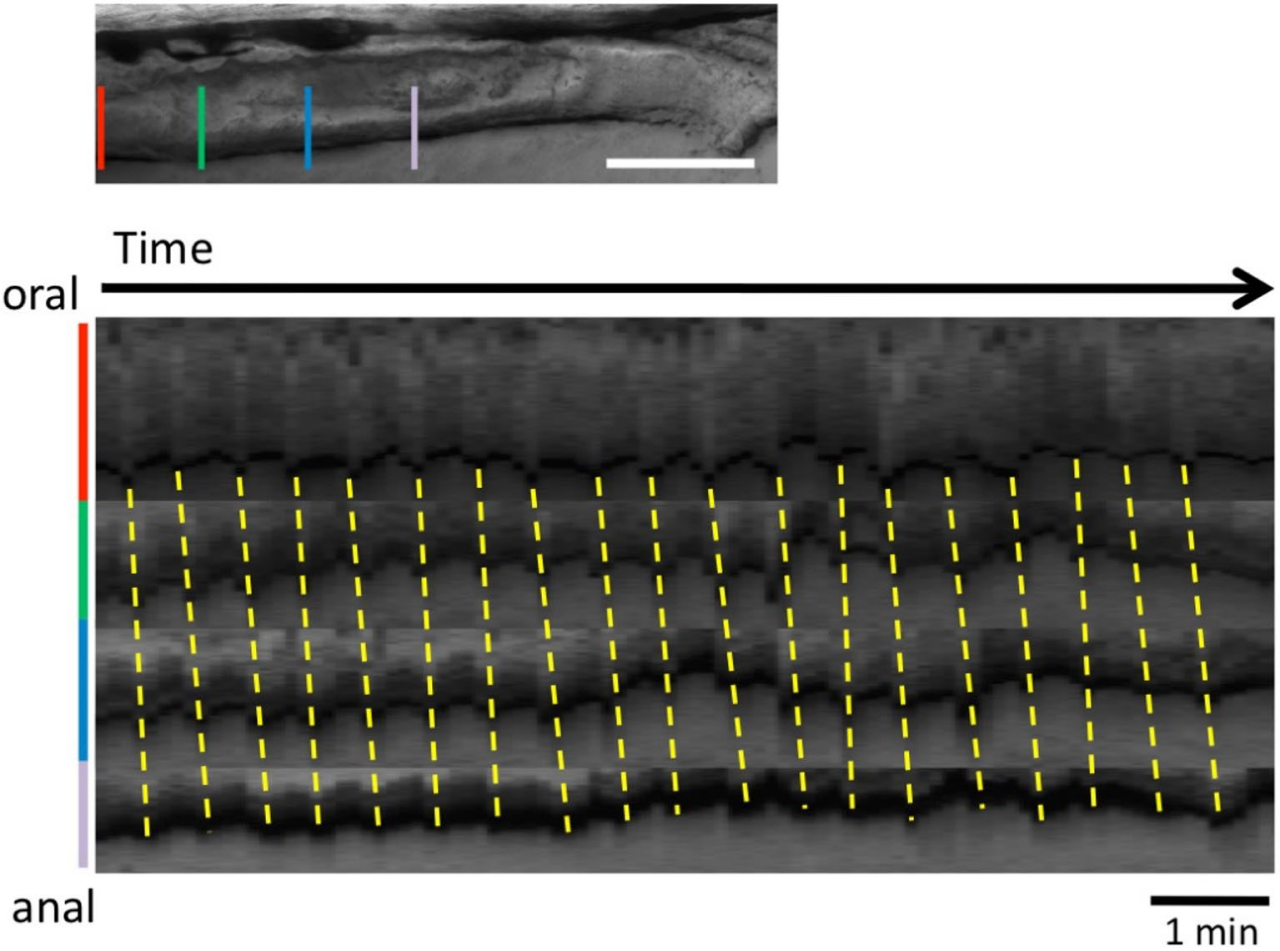
Posterior propagation of the regular movements, that is distinct from the peristaltic reflex, observed in the distal intestine of zebrafish larva at 8 dpf with a bolus. Kymographs of the bright field movie at four positions in the distal intestine (colored lines in the upper panel) along the anterior–posterior axis, from the data shown in Fig. 2 (20 min duration; Also see Supplementary video 1). Dotted lines demonstrate that the waves of periodic contractions propagate in the caudal direction. These regular movements may correspond to slow waves. See the text for the detail. Scale bar, 50 μm.

**Figure 5:**
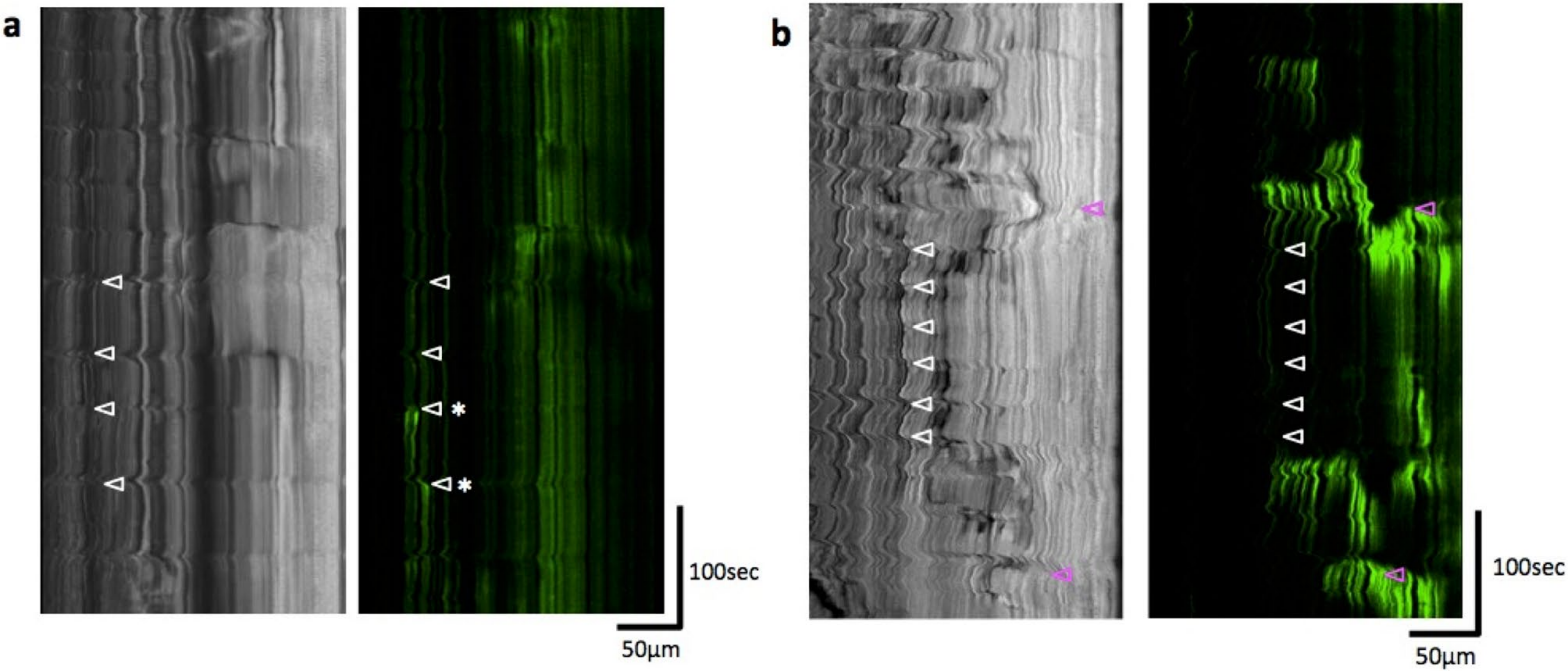
Regular movements observed in the distal intestine at 4 and 7 dpf and peristaltic reflex-like activity observed at 7 dpf without feeding. Kymgraphs of the bright field and GCaMP3 fluorescence movies, at 4 dpf (**a**) and 7 dpf (**b**). Regular movements (white triangles) were not always associated with Ca^2+^ events in the circular smooth muscles, although weak Ca^2+^ events could be sometimes associated with the regular movements, as indicated by asterisks in (**a**). On the other hand, peristaltic reflex-like movement (magenta triangles) which was sometimes observed without feeding was associated with strong Ca^2+^ events in the circular smooth muscles.

The kymograph analysis of the gut movement, in the bright field in both fed and unfed larvae, demonstrated weaker movement with regular and high frequency, whether with bolus (Fig. 4) or without (Fig. 5). Ca^2+^ signals in the circular muscles associated with this movement were weak or not detected (Figs. 2d, 5). These movements propagated caudally (4 dpf : 1.4 ± 0.1 cycles/min, 7 dpf : 1.7 ± 0.5 cycles/min, 8 dpf : 1.14 cycles/min v = 3.20 ± 1.22 μm/sec), which may correspond to the slow waves. They are distinct from peristaltic reflex or strong spontaneous ‘peristaltic reflex-like’ movement, either of which is associated with strong Ca^2+^ events with slower cycles.

**Figure 6:**
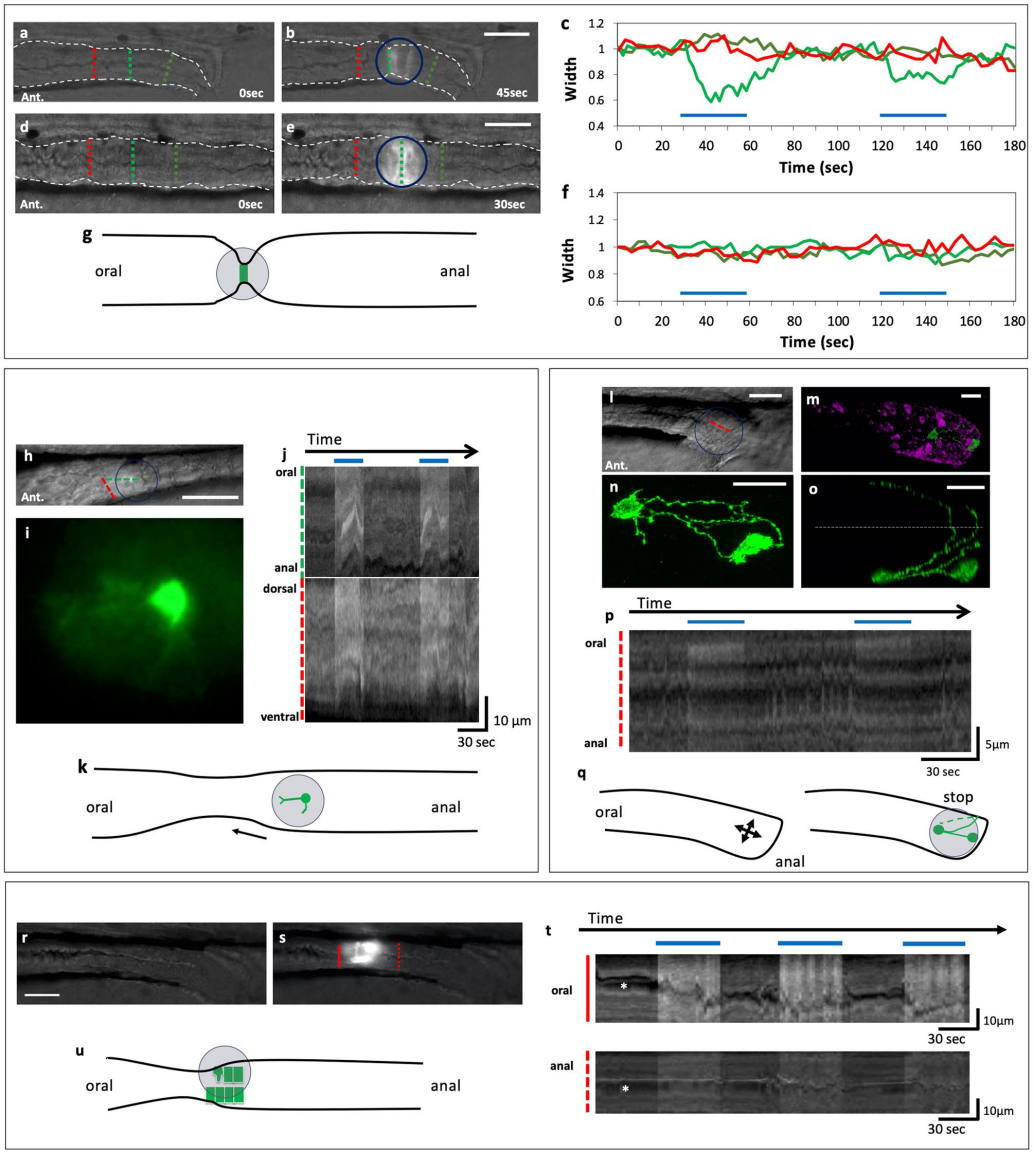
Optogenetic activation of a small number of smooth muscle cells, neurons or endodermal cells could induce or arrest gut movement. (**a–g**) Activation of circular smooth muscles caused local contraction of the gut. Blue-light irradiation (indicated by a circle) of circular muscles expressing ChR2-eYFP in SAGFF(LF)134A; Tg(UAS: ChR2-eYFP) (**a**,**b**) and the control expressing GFP in SAGFF(LF)134A; Tg(UAS: GFP) (**d**,**e**) in the posterior gut at 8 dpf. The changes in the width of the gut (**c**,**f**). Green lines indicate the position of the measurement in the spot of blue light; red lines at the oral side of the spot; yellowgreen lines at the anal side. Blue lines in (**c**,**f**) indicate the periods when blue light was applied. Schematic representation of the gut movement induced by the spot light (**g**). Lateral views. Ant., anterior. Scale bars, 50 μm. Also see Supplementary videos 3 and 4. (**h**–**k**) Activation of a single neuron could induce contraction at the oral side of the neuron. Live view of the midgut (**h**) and the neuron expressing ChR2-eYFP in a spot of the blue light, extending one of the neurites anteriorly (**i**) and two kymographs of (**g**) at the oral-to-anal (green) and the dorsal-to-ventral (red) dashed lines (**j**). Schematic representation of the gut movement induced by the spot light (**k**). Scale bars, 50 μm for h and 10 μm for i. Also see Supplementary video 5. (**l–q**) Activation of two neurons located near the anus could cause the arrest of the local gut movement at 8 dpf of Tg(hsp70: Gal4); Tg(UAS: ChR2-eYFP). Live image of the gut (**l**). Ant., anterior. Double immunostaining for anti-GFP indicating the cells expressing ChR2-eYFP (green) and anti-5-HT (magenta) (**m**). Enlarged views of these neurons at lateral view (**n**) and dorsal view (**o**) indicating that some axons are extending across the dorsal midline of the gut. Dotted line indicates the dorsal midline. Kymograph at the dashed red line in (**l**). Schematic representation of the arrest of the gut movement induced by the spot of blue light (**q**). Scale bars, 20 μm. Also see Supplementary video 6. (**r–u**) Activation of cells in the endodermal cell layer could induce a peristaltic reflex-like movement (a local constriction of the gut and active movement at the oral part) at 5 dpf of Tg(hsp70: Gal4); Tg(UAS: ChR2-eYFP). The same gut before (**r**) and during (**s**) the irradiation with the spot of blue light. Kymographs at the solid and dashed red lines in (**s**, **t**). Schematic representation of the gut movement induced with the light (**u**). Asterisks indicate the lumen of the gut. Arrows indicate the movement of the gut. Scale bars, 20 μm. Also see Supplementary video 7.

To investigate the function of the circular smooth muscles in gut movement, we expressed ChR2, a cation channel that is open upon irradiation with blue light^15^, by generating double transgenic fish SAGFF(LF)134A; Tg(UAS:ChR2-EYFP)^21^. Blue-light irradiation at a small spot caused a local constriction of the gut and closure of its lumen. We did not observe contraction at the anal or oral adjacent region (Fig. 6a–g, Supplementary videos 3 and 4). To investigate the functions of other types of cells in the gut, we generated double transgenic fish Tg(hsp70: Gal4); Tg(UAS: ChR2-EYFP). Heat shock at 6 dpf induced mosaic expression of ChR2 in some neurons at 8 dpf (Fig. 6h–q). Figure 6h–k shows an example in which a single neuron was found in a spot of blue light that extended axons in the oral direction (Supplementary video 5). In this case, contraction of the gut was observed at the oral side of the spot. The neuron may correspond to the descending interneuron (‘3’ in Fig. 1g). In Fig. 6l–q, two neurons expressing ChR2-EYFP were found in the ENS near the anus (Supplementary video 6). These neurons extend axons crossing the dorsal midline. When these neurons were irradiated with a spot of blue light, the rapid movement (7.7 ± 1.5 cycles/min, n = 3) of the anus was arrested immediately. They may represent inhibitory motoneurons or interneurons that regulate the inhibitory motoneurons. Heat shock at 3 dpf induced mosaic expression of ChR2-EYFP at 5 dpf in a different set of cells including mucosal epithelial cells and bowling-pin-shaped enterochromaffin (EC: cell ‘1’ in Fig. 1f,g) or enteroendocrine cell-like cells. Irradiation at a small spot caused a local constriction of the gut and active movement at the oral part but not at the anal part, mimicking a peristaltic reflex-like movement (Fig. 6r–u, Supplementary video 7).

Our data represent the first example of Ca^2+^ imaging of gut smooth muscles in zebrafish larvae in vivo as well as photo-manipulation of gut behavior, and they show evidence of a peristaltic reflex. The regular constriction of the gut at about 1 cycle/min may correspond to the slow waves produced by ICCs, and they may also correspond to the anterograde constrictions previously observed by video analysis in the larval gut without bolus^22^. Expression of markers for ICCs was reported in myenteric and submucosal layers in zebrafish^23^. These results provide a foundation on which to study the function of muscular systems, ENS, and other cell types in vivo in controlling gut behaviors at the single-cell level in the vertebrates.

## Methods

### Zebrafish

Zebrafish were raised and maintained according to standard procedures^24^ and stage by day post-fertilization at 28.5 °C (dpf)^25^. All procedures reported herein were carried out with the approval of the Institutional Animal Care and Use Committee of the University of Hyogo. All experiments were performed in accordance with the fundamental guidelines for proper conduct of animal experiment and related activities of the Ministry of Education, Culture, Sports, Science and Technology in Japan.

### Tg(UAS:GCaMP3)

The GCaMP3 gene was cloned into the EcoRI site of pUS^26^, giving rise to pUGCaMP3. The DNA construct was injected with transposase mRNA into zebrafish embryos at the single-cell stage by the use of air pressure. The injected fish were screened by crossing with a Gal4 driver, Tg(dld: Gal4)^20^.

### Immunohistochemistry

Zebrafish larvae were anesthetized and fixed in 4% paraformaldehyde (PFA) for 2 h at room temperature (RT). The larvae were washed 3 × 30 min in distilled water^27^ and then were incubated for 1 h in blocking solution (2% normal goat serum, 1% bovine serum albumin, 1%dimethylsulfoxide, 0.1% Triton X-100 in PBS). The larvae were incubated overnight at RT in primary antibodies diluted in blocking solution. The primary antibodies used were mouse anti-GFP (1: 5000 dilution; Invitrogen), rabbit anti-5-HT (1: 4000 dilution; Sigma-Aldrich) and rabbit anti-Desmin (1:20 dilution; Sigma-Aldrich). The larvae were rinsed extensively in PBS with Triton X-100 (PBST) and incubated overnight at RT in secondary antibodies diluted in blocking solution. The secondary antibodies were Alexa Fluor 488 (1: 500 dilution; Life Technologies) or 564 (1: 500 dilution; Invitrogen). After rinsing in PBST, larvae were transferred to 50% glycerol in PBS.

### Photoconversion

SAGFF(LF)134A; Tg(UAS:Kaede) larval zebrafish at 5 dpf were anaesthetized with Tricaine (Sigma-Aldrich; 160 mg l^−1^) and mounted on a cover glass-bottomed culture dish in a drop of 1% low melting point (LMP) agarose (Sigma-Aldrich) in H_2_O. For the photoconversion, we used a confocal microscope (TCS SP8; Leica) with a glycerol emersion 63 × /1.30 lens. Photoconversion can be achieved by scanning with a 405 nm laser. To photoconvert in the local area, we use the region of interest (ROI).

### ***Ca***^***2***+^***imaging***

SAGFF(LF)134A; Tg(UAS: GCaMP3) larval zebrafish were anaesthetized with Tricaine and mounted on a cover glass-bottomed culture dish in a drop of 1% LMP agarose in H_2_O. The agarose applied to the anus was removed to expel the bolus. For the time-laps Ca^2+^ imaging of the smooth muscles (Figs. 2 and 5), we used a FV300 confocal microscope (Olympus) with a water emersion 20 × /0.75 or a water emersion 40 × /0.80 lens. Each z-projection image was obtained from 7 z-slices by 5 mm increment (Confocal aperture: 5) at 6 s interval, and the duration of each experiment ranged from 13 to 20 min. For the time-laps Ca^2+^ imaging of the larva expressing heat-shock induced GCaMP3 (Fig. 3), we used an SP8 confocal microscope (Leica) with a glycerol emersion 63x/1.30 lens. Each z-projection image was obtained from 16 z-slices by 5 mm increment at 12 s interval for about 8 min. Image post-processing was done with Image J.

### Optical stimulation

To take a pigment, zebrafish larvae were treated with 1-phenyl-2-thiourea (PTU; Sigma-Aldrich; 30 mg l^-1^) from 1 dpf. To enable imaging, larvae (8 dpf) were anesthetized with Tricaine and embedded in 1% LMP agarose. We used an AxioPlan2 microscope (Zeiss) with a water emersion 40 × /0.80 lens, equipped with a DP72 camera (Olympus). Each frame was taken at 1 s exposure using Olympus image acquisition software, DP2-BSW.

### Heat shock

For heat-shock treatment, larvae raised at 28.5 °C were put in 50 ml plastic tubes (Falcon) and incubated in a water bath at 37 °C for 30 min.

## Supplementary Information

Supplementary Information 1.

Supplementary Video 1.

Supplementary Video 2.

Supplementary Video 3.

Supplementary Video 4.

Supplementary Video 5.

Supplementary Video 6.

Supplementary Video 7.

## References

[1] Sasselli V, Pachnis V, Burns AJ (2012). The enteric nervous system. Dev. Biol..

[2] Furness JB (2005). The Enteric Nervous System.

[3] Li ZS, Furness JB (1993). Nitric oxide synthase in the enteric nervous system of the rainbow trout, Salmo gairdneri. Arch. Histol. Cytol..

[4] Costa M, Brookes SJH, Hennig GW (2000). Anatomy and physiology of the enteric nervous system. Gut.

[5] Brookes SJH (2001). Classes of enteric nerve cells in the guinea-pig small intestine. Anatom. Record.

[6] Olsson C, Holmgren S (2001). The control of gut motility. Comp. Biochem. Physiol..

[7] Ward SM, Burns AJ, Torihashi S, Sanders KM (1994). Mutation of the proto-oncogene c-*kit* blocks development of interstitial cells and electrical rhythmicity in murine intestine. J Physiol..

[8] Camborova P, Hubka P, Sulkova I, Hulin I (2003). The pacemaker activity of interstitial cells of Cajal and gastric electrical activity. Physiol. Res..

[9] Wallace KN, Akhter S, Smith EM, Lorent K, Pack M (2005). Intestinal growth and differentiation in zebrafish. Mech. Dev..

[10] Holmberg A, Olsson C, Holmgren S (2006). The effects of endogenous and exogenous nitric oxide on gut motility in zebrafish *Danio rerio* embryos and larvae. J. Exp. Biol..

[11] Uyttebroek L (2010). Neurochemical coding of enteric neurons in adult and embryonic zebrafish (*Danio rerio*). J. Comp. Neurol..

[12] Kuhlman J, Eisen JS (2007). Genetic screen for mutations affecting development and function of the enteric nervous system. Dev. Dyn..

[13] Nakai J, Ohkura M, Imoto K (2001). A high signal-to-noise Ca^2+^ probe composed of single green fluorescent protein. Nat. Biotechnol..

[14] Nagel G (2003). Channelrhodopsin-2, a directly light-gated cation-selective membrane channel. PNAS.

[15] Deisseroth K (2006). Next-generation optical technologies for illuminating genetically targeted brain circuits. J. Neurosci..

[16] Nakayama S, Ikenaga T, Kawakami K, Ono F, Hatta K (2012). Transgenic line with gal4 insertion useful to study morphogenesis of craniofacial perichondrium, vascular endothelium-associated cells, floor plate, and dorsal midline radial glia during zebrafish development. Dev. Growth Differ..

[17] Hatta K, Tsujii H, Omura T (2006). Cell tracking using a photoconvertible fluorescent protein. Nat. Protcol..

[18] Tian L (2009). Imaging neural activity in worms, flies and mice with improved GCaMP calcium indicators. Nat. Methods.

[19] Okamoto S, Nakagawa M, Hatta K (2013). Stochastic Ca^2+^ waves that propagate through the neuroepithelium in limited distances of the brain and retina imaged with GCaMP3 in zebrafish embryos. Zool. Sci..

[20] Scheer N, Riedl I, Warren JT, Kuwada JY, Campos-Ortega JA (2001). A quantitative analysis of the kinetics of Gal4 activator and effector gene expression in the zebrafish. Mech. Dev..

[21] Itoh M, Yamamoto T, Nakajima Y, Hatta K (2014). Multi-stepped optogenetics connects neurons and behavior. Curr. Biol..

[22] Holmberg A, Olsson C, Hennig GW (2007). TTX-sensitive and TTX-insensitive control of spontaneous gut motility in the developing zebrafish (Danio rerio) larvae. J. Exp. Biol..

[23] Rich A (2007). Kit-like immunoreactivity in the zebrafish gastrointestinal tract reveals putative ICC. Dev. Dyn..

[24] Westerfield M (1995). The zebrafish book: a guide for the laboratory use of zebrafish (Danio rerio).

[25] Kimmel CB, Ballard WW, Kimmel SR, Ullman B, Schilling TF (1995). Stages of embryonic development of the zebrafish. Dev. Dyn..

[26] Aramaki S, Hatta K (2006). Visualizing neurons one-by-one in vivo : optical dissection and reconstruction of neural networks with reversible fluorescent proteins. Dev. Dyn..

[27] Ungos JM, Karlstrom RO, Raible DW (2003). Hedgehog signaling is directly for the development of zebrafish dorsal root ganglia neurons. Development..

