# Supplementary material for: Ca2+-imaging and photo-manipulation of the simple gut of zebrafish larvae in vivo
Source: Sci Rep. 2022 Feb 7;12:2018. doi: 10.1038/s41598-022-05895-4 (PMC8821699; doi:10.1038/s41598-022-05895-4)
Supplement: Supplementary file 1 — Supplementary Information 1. [file 41598_2022_5895_MOESM1_ESM.pdf]

Supplementary Video Legends

Ca^{2+}-imaging and photo-manipulation of the simple gut of zebrafish larvae in vivo.

Shin-ichi Okamoto and Kohei Hatta

Graduate School of Science, University of Hyogo, 3-2-1 Kouto, Kamigori, Ako-gun, Hyogo 678-1297 Japan

Supplementary Video 1. Imaging of Ca^{2+} events in the circular smooth muscles during peristaltic movements in the gut of SAGFF(LF)134A; Tg(UAS: GCaMP3) embedded in agarose at 8 dpf. The speed is 50 times faster than real time. Also see Figure 2.

Supplementary Video 2. Imaging of Ca^{2+} events in a variety of cell types including putative enteric neurons and circular smooth muscles during peristaltic reflex in the gut of Tg(hsp70: Gal4); Tg (UAS: GCaMP3) embedded in agarose at 8 dpf. The speed is 50 times faster than real time. Also see Figure 3.

Supplementary Video 3. Optogenetic activation of the circular smooth muscles causes local constriction of the gut. Blue-light irradiation of circular muscles expressing ChR2-eYFP in the circular smooth muscles in the gut of SAGFF(LF)134A; Tg(UAS: ChR2-eYFP) embedded in agarose at 8 dpf. The speed is 5 times faster than real time. Also see Figures 6a-c, g.

Supplementary Video 4. The control experiment for the Supplementary Video 3. Blue-light irradiation of circular muscles expressing GFP has no effect on the gut of SAGFF(LF)134A; Tg(UAS: GFP) embedded in agarose at 8 dpf. The speed is 5 times faster than real time. Also see Figures 6d-f.

Supplementary Video 5. Optogenetic activation of an enteric neuron induces contraction at the oral side of the neuron in the gut of Tg(hsp70: Gal4); Tg (UAS: ChR2-eYFP) embedded in agarose at 8 dpf. The speed is 5 times faster than real time. Also see Figures 6h-k.

Supplementary Video 6. Optogenetic activation of two enteric neurons located near the anus causes the arrest of the local gut movement in Tg(hsp70: Gal4); Tg (UAS: ChR2-eYFP) embedded in agarose at 8 dpf. The speed is 5 times faster than real time. Also see Figure 6l-q.

Supplementary Video 7. Optogenetic activation of mucosal epithelial cells including bowling-pin-shaped enterochromaffin cell-like cells causes a local constriction of the gut and active movement at the oral part but not at the anal part, mimicking a peristaltic reflex in the gut of Tg(hsp70: Gal4); Tg (UAS: ChR2-eYFP) embedded in agarose at 5 dpf. The speed is 5 times faster than real time. Also see Figures 6r-u.
